# Rapid single nucleotide polymorphism mapping in *C. elegans*

**DOI:** 10.1186/1471-2164-6-118

**Published:** 2005-09-12

**Authors:** M Wayne Davis, Marc Hammarlund, Tracey Harrach, Patrick Hullett, Shawn Olsen, Erik M Jorgensen

**Affiliations:** 1 Department of Biology, University of Utah, Salt Lake City, Utah 84112-0840, USA

## Abstract

**Background:**

In *C. elegans*, single nucleotide polymorphisms (SNPs) can function as silent genetic markers, with applications ranging from classical two- and three-factor mapping to measuring recombination across whole chromosomes.

**Results:**

Here, we describe a set of 48 primer pairs that flank SNPs evenly spaced across the *C. elegans *genome and that work under identical PCR conditions. Each SNP in this set alters a *Dra*I site, enabling rapid and parallel scoring. We describe a procedure using these reagents to quickly and reliably map mutations. We show that these techniques correctly map a known gene, *dpy-5*. We then use these techniques to map mutations in an uncharacterized strain, and show that its behavioral phenotype can be simultaneously mapped to three loci.

**Conclusion:**

Together, the reagents and methods described represent a significant advance in the accurate, rapid and inexpensive mapping of genes in *C. elegans*.

## Background

Single Nucleotide Polymorphism (SNP) mapping has transformed studies of genetic linkage in *C. elegans *since its introduction in 2001[1]. In SNP mapping, DNA sequence polymorphisms between the wild-type *C. elegans *strain (N2 Bristol) and a closely related strain (CB4856 Hawaiian) are used as genetic markers. Compared to other markers that have been used for genetic mapping, SNPs have two distinct advantages. First, unlike conventional marker mutations that cause visible phenotypes, SNPs in general have no associated phenotype. Thus, mutant phenotypes that are masked by conventional marker mutations, such as those with subtle behavioral defects, can be mapped using SNPs. Second, SNPs are far denser than other markers, including both visible markers and DNA polymorphisms such as Tc1 insertions. Because SNPs are approximately as dense as genes, SNP mapping can in theory provide single-gene resolution [2]. Together, these two advantages have made SNP mapping the technique of choice for many *C. elegans *researchers.

SNP mapping is usually done in two phases. The first phase, chromosome mapping, is similar to traditional two-factor mapping and seeks to identify the relevant chromosome and rough position of the gene of interest. The second phase, interval mapping, seeks to place the gene of interest in an interval between two SNPs, and can be used iteratively to fine map the gene. SNP detection in both phases is typically performed by using only SNPs that alter a restriction site, which are also known as snip-SNPs [1]. Although other SNP detection methods have been used for mapping in *C. elegans*, such as fluorescence polarimetry and indel detection [3,4], snip-SNPs are attractive because they require low initial investment and do not require specialized equipment. However, snip-SNP detection requires PCR amplification of the SNP region, digestion with the appropriate restriction enzyme, and gel electrophoresis. These multiple steps can be daunting, particularly during chromosome mapping when many SNPs need to be assayed simultaneously.

The method described here streamlines the procedure for detecting snip-SNPs, making faster, more efficient mapping possible. First, we identified 48 SNPs that met our criteria (8 per chromosome). This means that every part of the genome is linked to multiple SNPs, so that adjacent SNPs serve as internal controls, and also that sub-chromosome position can be determined. Second, we simplified the PCR step by identifying primer sets that all work at identical amplification conditions, and that are tolerant of being added to reactions by pin-replication. This enabled us to quickly amplify across 48 snip-SNPs in a single 96-well PCR plate. Third, we streamlined restriction digestion by using only snip-SNPs that can be distinguished by a single restriction enzyme, *Dra*I. This enzyme is relatively inexpensive and tolerant of PCR buffer salts. This allowed us to perform digestion in the original PCR plate, by adding to each well an identical digestion cocktail. Finally, primer locations were designed so that the informative digestion products can be resolved on an agarose gel. The accessibility of this mapping procedure, together with its speed, low cost, robustness, and accuracy, should make it a preferred option for most *C. elegans *labs.

## Results and discussion

### Chromosome mapping

**Figure 1:**
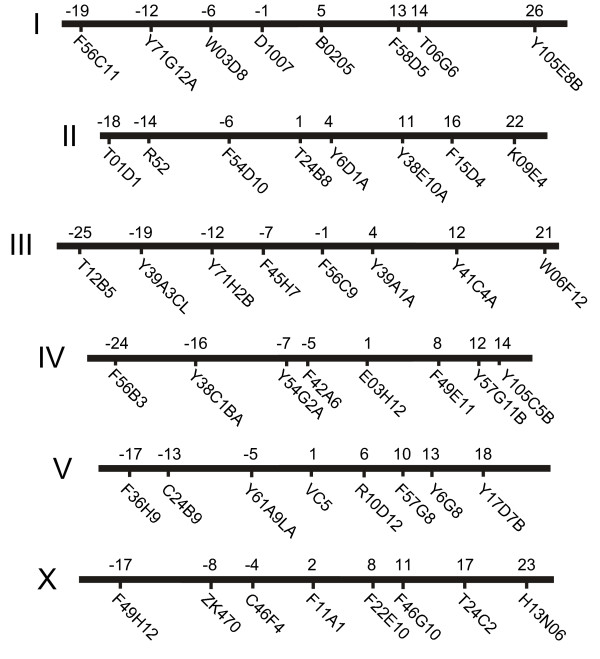
*Genetic position of SNPs used for mapping*. The genetic position of each SNP was obtained from Wormbase Release 143.

To facilitate mapping a mutation onto a chromosome, we designed a set of PCR reagents based on modifications of the principle of bulk segregant analysis described by Wicks et al. [1]. Our primary goal was to simplify the procedure by performing all steps in a 96-well format PCR plate, and designing each SNP reaction to be performed under identical conditions. To design a set of primers that can be used for SNP mapping in a 96-well format, two conditions must be met: the primers must all use the same conditions for polymorphism detection, and the primers must all use the same conditions for amplification. First, we simplified SNP detection by using only SNPs that could be detected using a single restriction enzyme. Since SNPs are concentrated in non-coding A/T-rich regions of the genome, we reasoned that good coverage would be obtained from the enzyme *Dra*I, which recognizes the sequence TTT^AAA. We identified all *Dra*I SNPs in a custom database (available as supplementary material) that incorporated all SNPs identified by the Genome Sequencing Center, Washington U, St. Louis, MO (6,333 total SNPs, 248 *Dra*I SNPs in our database) [5] and by Exelixis, South San Francisco, CA (9,295 total SNPs, 257 *Dra*I SNPs in our database) [3]. From among these we selected eight candidate *Dra*I SNPs on each chromosome that were far enough from nearby *Dra*I sites (typically >200 bp on one side, >50 bp on the other) to enable detection of cleavage at the SNP *Dra*I site. The genetic positions of the resulting 48 *Dra*I SNPs are shown in Figure 1.

Next, to enable simultaneous amplification of all selected SNPs in a 96-well format, we chose primer pairs with similar annealing temperatures and product length. The program Primer3 [6] was used to design primers to amplify short sequences (typically 300–500 bp) containing each selected *Dra*I SNP. Optimum T_m _was set to 60°C. Primer pairs were tested, and unsatisfactory pairs were redesigned until all 48 primer pairs amplified robustly in simultaneous PCR reactions in a single plate. The resulting primer sequences are shown in Table 1.

**Table 1:** Dra*I SNP primers, locations and band sizes*. In each pair of primers, the left primer is listed first; all primer sequences are given 5' to 3'. Interpolated genetic positions are from [9] release WS143.

Genetic Location	Physical Location	Clone	N2 digest	CB4856 digest	Primers	Wormbase Identifier
I, -19	169, 017	F56C11	354, 146	500	ATGCCAGTGATAAGGAACGG	snp_F56C11 [4]
					TCACATCCCTTGTCGATGAA	
I, -12	1,905,969	Y71G12A	503, 72	377, 126, 72	TCGAAATCAGGGAAAAATTGA	snp_Y71G12 [3]
					ACGATTTTCGGGGAGTTTTT	
I, -6	2,818,973	W03D8	395, 144	538	GTTTTCACTTTTGCCGGTGT	pkP1052
					TGAAGGCGCATATACAGCAG	
I, -1	4,594,014	D1007	325, 134, 41	459, 41	AAAATATCAGGAAAGAGTTTCGG	snp_D1007 [7]
					TTTAAAGATTAAGGGTGGAGCG	
I, 5	10,722,146	B0205	494	365, 129	ATCTGGCACCAAATATGAGTCG	CE1-247
					AATCTCGATTTTCAAGGAGTGG	(rs3139013)**
I, 13	12,047,594	F58D5	445	295, 151	TCCTGGATAATCCCCAAAAA	snp_F58D5 [4]
					CCCTGCCATTGATCTTGTTT	
I, 14	12,729,812	T06G6	236, 99, 78	335, 78	TTGAAATCCCCTTTAAAATCCC	uCE1-1361
					ACACTGGGTACCTGACTCATGC	
I, 26	14,682,016	Y105E8B	360, 114, 27	474, 27	ATTATTAACGGCCACGGTGA	snp_Y105E8B [3]
					CCCACACACTCTCACCTTCA	
II, -18	176,720	T01D1	263, 112	375	CCGAATTTTCAAATGGATGC	pkP2101
					CCATTGGAATTGCACACAAA	
II, -14	2,121,018	R52	345	236, 109	CTGTGCTGTTGACGATATTGG	snp_R52 [5]
					ATGTCTCATTGCAAAATTCGG	
II, -6	3,828,599	F54D10	516	387, 129	TTGTGAGCTTATATCTCAGTTGTCG	pkP2103
					AGATTTGGTTAGAAATATCACCGC	
II, 1	9,052,466	T24B8	373, 121	494	TCAAAAACTTACAATCAATCGTCG	snp_T24B8 [1]
					CCAGAAAATCTGCACAGAAGG	
II, 4	11,827,835	Y6D1A	224, 117, 124, 44	340, 124, 44	TTCTTCAAAAAGTCTAGGTTCAGCA	snp_Y6D1 [1]
					GGGGACGAAAACGGAGTTTG	
II, 11	12,605,350	Y38E10A	483	352, 132	ACCGTTTAATAGGATTATTTGGG	uCE2-2131
					AAGTCTGCGGAATAATTGATGG	
II, 16	13,235,564	F15D4	500	368, 132	TTCCAGGTAATACACATACAACTCC	pkP2116
					AAAAACACAAAGTTCAAAAACCC	
II, 22	14,132,466	K09E4	365, 119	484	CCACTGGCTATAAGCTTTTCTAGG	CE2-215
					TAAGGATTTCAGGCTTTTAGGC	(rs3139227)**
III, -25	939,698	T12B5	206, 189	395	TATCATCGAAATCCCGGAAA	uCE3-637
					TTCGGACGGGAGTAGAATTG	
III, -19	1,827,732	Y39A3CL	342, 78, 76	272, 78, 76, 70	TCCCAATTTCCCTCTAAAAACC	uCE3-735
					TTGAATTTGGACCATTTTGAGG	
III, -12	2,599,699	Y71H2B	368, 105	473	GAGGAACCAAATCTGGCGTA	snp_Y71H2B [2]
					TGAAAACTTGGAAAATCGGTG	
III, -7	3,359,033	F45H7	239, 85, 27	196, 85, 43, 27	AATTTGAATCAGTGACTTTTGGC	CE3-127
					TTTCTGCAAACATTTTTCTTCG	(rs3139272)**
III, -1	7,320,107	F56C9	486	354, 132	AAAAATACATGTCTACACAACCCG	snp_F56C9 [1]
					TTTCTTATCACTGTGCAGTCTTACC	
III, 4	10,652,476	Y39A1A	355, 142, 30	497, 30	AGCGTTAAAGTATCGGTTATTTCG	snp_Y39A1 [9]
					TAAATTCATTTCAAACAATCGAGC	
III, 12	11,656,188	Y41C4A	339, 156	495	ATCAAGTTTCTGATTGCTCTTTCC	snp_Y41C4 [2]
					AAAAACGTGATTTTTCAATTTTGC	
III, +21	13,715,622	W06F12	273, 137, 78	200, 137, 78, 73	AGCAGGCTCACCATCATCATCA	uCE3-1426
					GACATTACGGTAGAGGAGATGGA	
IV, -24	795,461	F56B3	301, 128, 71	429, 71	TGATGGTGTGTCTGCGTACC	uCE4-515
					AGAGCTGGAGAGCACGGATA	
IV, -16	1,799,032	Y38C1BA	187, 304	491	CGCATAAATCCAACGTTCTCTG	snp_Y38C1B [2]
					AATCCATAAGTTTCGTGTTGGG	
IV, -7	2,761,525	Y54G2A	498	250, 248	ACTCGGCATCCTCACGC	snp_Y54G2 [5]
					GTTGAAAATTTTTTCATAGCTATCATC	
IV, -5	3,347,952	F42A6	295, 124	419	TGCTGAAATATTGGAAAATTGAGG	pkP4055
					TTATATCGTCGAGGAGGTTAGAGG	
IV, 1	4,991,851	E03H12	376	300, 76	AAAATGGGAAGCGTACCAAA	pkP4071
					TGCTTGTAGCGTTTCCAAGA	
IV, 8	13,049,020	F49E11	313, 77	390	GACACGACTTTAGAAACAACAGC	snp_F49E11 [1]
					TGGTATGGAGTCCCTATTTTGG	
IV, 12	14,566,396	Y57G11B	284, 162, 52*	327, 119, 52*	TGTAAATACCCCACATTTCAAGC	snp_Y57G11B [2]
					AAATTTCCAATTGTTCAAAGCC	pkP4095*
IV, 14	16,085,085	Y105C5B	241, 108, 78, 48	319, 108, 48	TCGAATTGTTGTGTTTCTTTTGA	pkP4099
					TTCCAATTTTCTCGGTTTGG	
V, -17	1,773,464	F36H9	307, 87, 79	386, 87	TTTCGGAAAATTGCGACTGT	pkP5076
					CGCGTTTTGGAGAATTGTTT	
V, -13	2,726,662	C24B9	288, 167	455	TCATCTGTTATTTCGTCTCTTGC	uCE5-828
					CGGTAATAATATGCTTTGTGGG	
V, -5	4,550,757	Y61A9LA	454	307, 147	GAGATTCTAGAGAAATGGACACCC	snp_Y61A9L [1]
					AAAAATCGACTACACCACTTTTAGC	
V, 1	7,089,411	VC5	435, 70	300, 135, 70	AGAAATGATCCGATGAAAAAGC	pkP5097
					CCGATAGTGTTCATAGCATCCC	
V, 6	13,951,850	R10D12	500	348, 152	CAAATTAAATATTTCTCAAAGTTTCGG	***
					ACATAAGCGCCATAACAAGTCG	
V, 10	16,321,481	F57G8	475	288, 187	TAAAGCCGCTACGGAAATACTC	pkP5129
					ATTTTCTCCCTAATTCCAGGTG	
V, 13	17,610,508	Y6G8	282, 205	487	CATTCATTTCACCTGTTGGTTG	uCE5-2609
					TCGGGAAGATAATCAAAATTCG	
V, 18	18,782,547	Y17D7B	324, 164	488	GAAATTCAAATTTTTGAGAAACCC	snp_Y17D7B [3]
					TTCAGACCATTTTTAGAATATTCAGG	
X, -17	2,065,464	F49H12	540	321, 219	ATATGTGAGTTTACCATCACTGGG	pkP6143
					ACGTTTTGAAAAATTTGGTTGC	
X, -8	4,161,493	ZK470	422, 72, 40	326, 96, 72, 40	CCAAAACGGCCAAGTATCAG	pkP6105
					TTGCACTCTTTCTCCTTCCG	
X, -4	5,934,688	C46F4	169, 54, 51, 35, 22	223, 51, 35, 22	AAGTGTTCAATGATTTTGTCTAATTG	uCE6-981
					TGACAGGAGAATACTTTTGAAGG	
X, 2	10,637,922	F11A1	409, 133	542	AGCAACAAACAATGCAACTATGG	snp_F11A1 [2]
					TAAACAAGAGGGTACAAGGTATCG	
X, 8	12,750,713	F22E10	341, 126	467	TTAAAACCATACAATTCTTCTCAGC	snp_F22E10 [1]
					GAATTCCCAATCAACAGAGAGC	
X, 11	13,339,566	F46G10	318, 191, 37	509, 37	ACTGTTTACCGCGTCTTCTGC	pkP6132
					CCGTGTATATAAGAAAATGTGTTCG	
X, 17	14,547,382	T24C2	409, 34	302, 107, 34	GCTGGGATTTTGAAGAGTTGTT	uCE6-1459
					CAGTGAATCATCCGTTGAATTT	
X, 23	15,500,013	H13N06	358, 134	492	CAAATACCAAAGTTGATCGTGG	uCE6-1554
					TTGTTGCAATTAAATCAAACGG	

*Y57G11B has two DraI SNPs within a single PCR product.

**dbSNP IDs are given where the SNP has been submitted to the NCBI dbSNP database [10].

***The SNP on R10D12 has not been added to wormbase. It can be found at [11]

**Figure 2:**
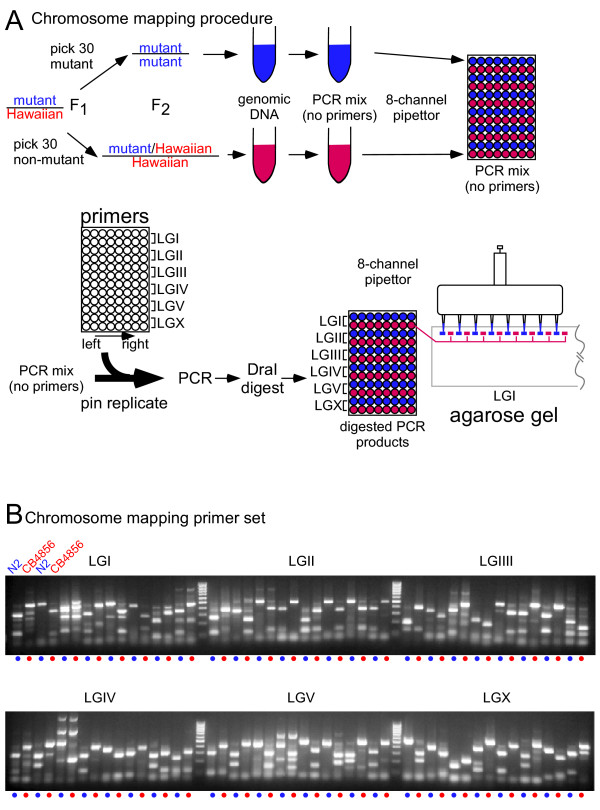
*Procedure for chromosome mapping*. (A), Method. Typically 30 mutant animals (homozygous Bristol DNA surrounding the mutation) and 30 wild-type animals (heterozygous Bristol/Hawaiian or homozygous Hawaiian DNA) are lysed in 20 μL lysis buffer. The lysate is then added to a PCR mix lacking primers, and the mix is aliquoted into every other row of a 96 well plate. Primers are added by pin replication from a master plate. Because the 8-channel pipette loads every other lane of the gel, each mutant reaction is placed next to its control. DNA ladder is typically placed in lanes 17 and 34. (B), Results from homozygous N2 Bristol and CB4856 Hawaiian genotypes. 50 Bristol adults and 50 Hawaiian adults were lysed in 20 μL lysis buffer, and used for the DNA template for the 48 PCR reactions covering all six chromosomes. Note that pure Bristol and Hawaiian DNA was used for each PCR reaction in the gel shown. When mapping a recessive mutant in the Bristol background against the Hawaiian strain, unlinked SNPs will display a 50-50 mix of Bristol bands and Hawaiian bands in both mutant and non-mutant lanes. Linked SNPs will display an enrichment of Bristol bands in the mutant lane, approaching 100% Bristol for tight linkage. The non-mutant lane will display a 2/3 to 1/3 enrichment of Hawaiian compared to Bristol DNA.

Finally, we devised a set of procedures that maximize speed and minimize the potential for error during reaction set up and gel loading (Figure 2). Our chromosome mapping procedure begins with the same genetic manipulations as other SNP mapping protocols. Hawaiian males are crossed into the mutant strain to produce heterozygous F1 animals. Homozygous F_2 _animals from the heterozygous F1 animals are identified based on their mutant phenotype. At the same time, animals with a non-mutant phenotype, which are enriched for Hawaiian sequences at the locus of interest, are also isolated (Figure 2). Thirty to fifty animals of each class are combined into two tubes and lysed using detergent and proteinase K (Figure 2). A PCR master mix, *not *including PCR primers, is then assembled and added to each lysate. These PCR mixes are then dispensed into alternating rows of a 96-well PCR plate using a multi-channel pipettor (Figure 2). These three simple steps generate a set of 96 PCR master mixes ready for the final addition of primers.

Because there are 96 separate reactions, each requiring addition of a specific primer pair, we generated a pre-arrayed set of primers, which are then added to the PCR master mixes by pin replication. Primer pairs described above are arrayed in pairs into a microtiter plate at 10 μM each primer ('primers' Figure 2A), with each row containing the primer pairs for the eight SNPs along a single chromosome. Adjacent rows contain a duplicate set of primers for a particular chromosome, and the plate of primers is pin-replicated into the master PCR mix. After amplification, PCR products are digested in the plate with *Dra*I in a final volume of 15 μL and loaded onto a 2.5% agarose gel using an 8-channel pipette. Because we use a gel comb with wells spaced half the distance between pipette tips of the multi-channel pipette, we can automatically load the mutant samples from the upper row and wild-type samples from the lower row for each SNP pair in adjacent wells. The resulting gel displays all 48 SNP markers, from left to right and from chromosome I to X (Figure 2B). Each mutant SNP is next to its non-mutant control, so that the whole genome can be quickly scanned for linkage.

**Figure 3:**
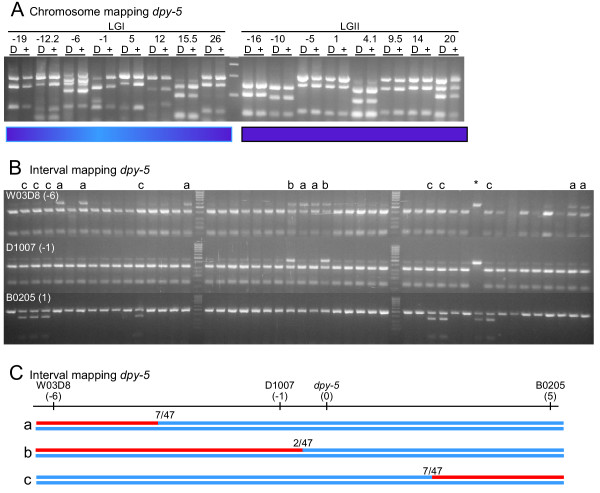
*SNP mapping *dpy-5(e61). A, chromosome mapping. Each pair of lanes shows results from the SNP at the indicated genetic map position, using either the Dumpy (D) or the wild-type (+) template. Linkage is visible as an increase in the proportion of Bristol N2 DNA in Dumpy lanes compared to the wild-type lanes, and is visible on LGI from -12.2 to 15.5. *B and C, interval mapping*. Each column in B is an individual Dpy recombinant, assayed for the three SNPs W03D8 (top row), D1007 (middle row), and B0205 (bottom row). Most (31/47) recombinants show Bristol DNA at all three SNPs. This indicates that these recombinants were homozygous Bristol at these loci, as expected for tightly linked markers. Sixteen animals show half Bristol and half Hawaiian DNA at one or more loci, indicating that they have one chromosome that is recombinant in this interval. Columns marked "a" are recombinant in the W03D8-D1007 interval, those marked "b" in the D1007-*dpy-5 *interval, and those marked "c" in the *dpy-5*-B0205 interval. These data are summarized in C, which depicts the three recombinant genotypes using blue for Bristol DNA and red for Hawaiian DNA. One recombinant, marked with an asterisk, is homozygous Hawaiian at two SNP loci and heterozygous at the third, and is thus very unlikely to be homozygous Bristol at the *dpy-5 *gene (see Results for explanation).

To validate the final set of primers for chromosome mapping experiments, we used them to map *dpy-5*, a mutation with a well known genetic position. We crossed CB4856 Hawaiian males to a triply-marked mapping strain, EG1000 *dpy-5(e61) I; rol-6(e187) II; lon-1(e1820) III*. We allowed the heterozygous F1s to self, and from the F2 generation we picked 50 Dpy and 50 non-Dpy animals into separate lysis reactions. We performed chromosome mapping PCR on these lysates using primer sets from LGI and LGII (Figure 3A). As expected, we found linkage to the center of LGI and no linkage to LGII.

### Interval mapping

**Figure 4:**
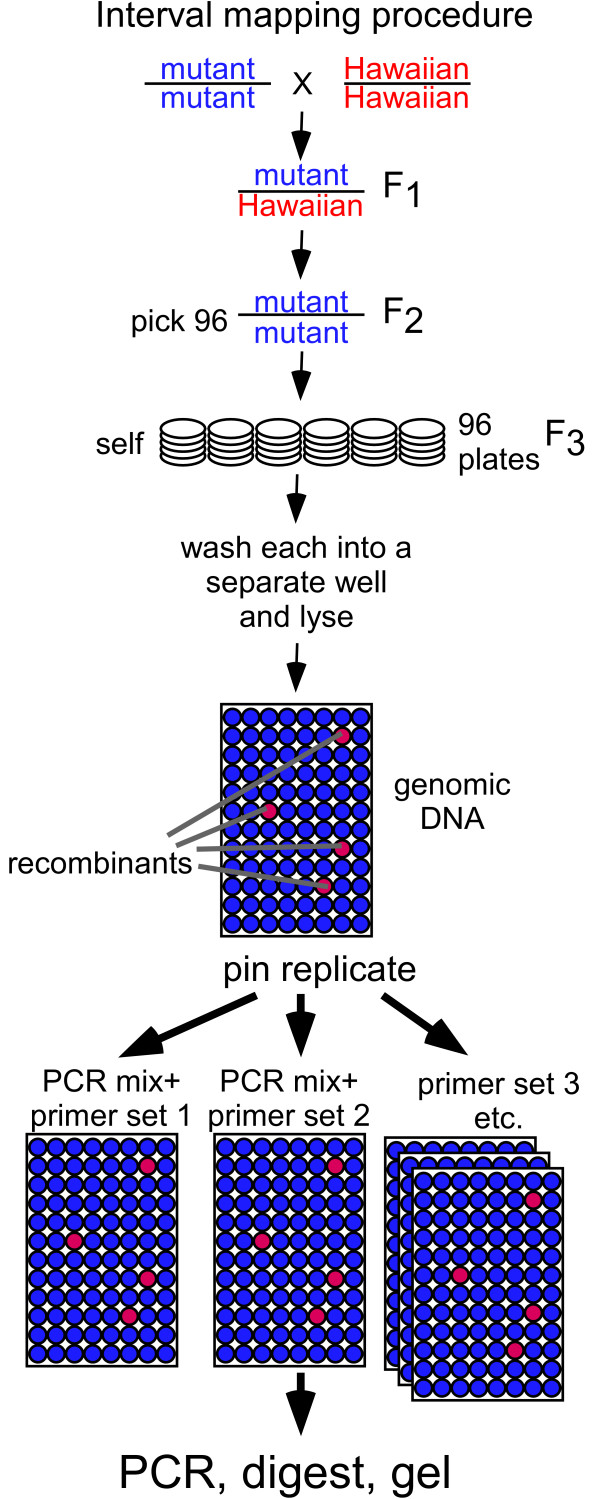
*Interval mapping*. Individual recombinants are singled from heterozygotes and the animal, or a representative sample of their progeny, are placed in wells of a 96-well PCR plate and lysed. The plate may also contain three control wells, with Bristol, Hawaiian, and a 50-50 mix of animals. DNAs from the lysed animals are pin-replicated into a PCR master mix containing primers for the desired SNP. The plates are processed for PCR amplification, digested with *Dra*I and samples run on an agarose gel.

After determining the rough position of a mutation on a chromosome using chromosome mapping, mutations can be quickly mapped to a genetic interval using the same efficiencies of the 96-well format employed in chromosome mapping. Interval mapping differs from chromosome mapping in that the genotype of individual mutant animals, rather than the genotype of pooled animals, must be determined. Also, it is necessary to assay these mutant DNAs for many SNPs within the interval for which linkage has been established. Therefore, it is most convenient to pin replicate the DNA templates, rather than the primers (Figure 4).

Briefly, we crossed Hawaiian males into our mutant strain (isolated from Bristol N2) to generate a heterozygous strain. From the progeny of these heterozygotes, we singled 96 mutant animals onto worm growth plates and allowed them to lay self-fertilized embryos. After the F3 progeny had grown to the adult stage, we washed about a quarter of the F3 progeny from the plate into individual wells of the 96-well plate (see Methods). Deriving templates from the self progeny of a homozygous mutant has the advantage of allowing each mutant recombinant to be scored as a population, rather than a single animal. Also, having additional template is convenient for additional rounds of PCR if higher resolution is desired. We found that 96-well plates containing the lysed worms can be frozen at -80°C and reused successfully after many rounds of thawing and re-freezing. If more rapid mapping was required, we found it possible to remove the single F2 animal (after it had laid sufficient embryos to ensure propagation of the strain) and to lyse it for analysis of its SNPs. Specifically, a drawn out and sealed Pasteur pipette was used as a pick to place each F2 adult individually into 5 μL of lysis buffer in a 96-well plate. The embryos on the worm plate could later be used as a source of DNA for additional mapping experiments.

Lysed DNA from each well was pin replicated into 96-well PCR plates containing complete PCR cocktail minus template. Each plate included the primer set for a single SNP. Typically we use plates representing four adjacent SNPs from the section of a particular chromosome that had shown linkage in the chromosome mapping experiments. For each plate, PCR, digestion and gel electrophoresis were performed as for chromosome mapping.

To validate the technique for mapping a mutation to an interval, we once again used the previously mapped gene *dpy-5*. We crossed Hawaiian males into the strain EG1000 as described above, and allowed the heterozygous progeny to self-fertilize. From the F2 progeny, we singled 48 Dpy animals onto individual plates. When these plates had starved, we washed each population of progeny into a well containing lysis buffer in a 96-well plate. The lysed DNA was assayed using three SNP primer pairs from the center of LGI (Figure 3B). Since each well contained progeny of a single F2 animal, it was possible to determine whether that animal was homozygous Bristol, homozygous Hawaiian, or heterozygous Bristol/Hawaiian at each SNP. From these data we could identify *dpy-5*-containing Bristol chromosomes that have recombined with Hawaiian DNA to the left of *dpy-5 *(recombinant types 'a' and 'b', Figure 3B and 3C), and to the right of *dpy-5 *(recombinant type 'c', Figure 3B and 3C). Keep in mind that each worm contains two *dpy-5 *containing chromosomes, but at regions near the mutation, usually only one is recombinant as illustrated in Figure 3C. We found, as expected, that our map data placed *dpy-5 *between -1 and 5 on LGI at approximately 0.3, very close to the known map position of 0.0 for *dpy-5 *(Figure 3C). Interestingly, one of the 48 Dpy F2 animals showed no linkage to LGI (see * in Figure 3B). The plate of worms that had been used to generate that lysate was chunked onto a new plate to verify the Dpy phenotype. Surprisingly, they were Dpy, but less so than *dpy-5 *homozygotes. We have observed that this Dpy phenotype segregates at a low frequency from several unrelated crosses between Hawaiian CB4856 and Bristol N2. Indeed, the plate segregated Rol-6 animals that were also Dpy. This confirms that the phenotype is not due to a *dpy-5 *mutation, since *dpy-5 *is epistatic to *rol-6*, but apparently the synthetic Dpy phenotype is not.

### Mapping unknown mutations

To illustrate the utility of these methods we mapped a suppressor mutation of a behavioral phenotype. The map data demonstrate that these methods were able to map the original uncoordinated mutation and two other loci that synthetically suppress it in a single experiment. This strain, KY5029, was isolated in a screen for suppressors of *unc-31(e928) *(gift of Liakot Khan and Kouichi Iwasaki). *unc-31 *encodes the *C. elegans *homolog of CAPS, a protein required for dense-core vesicle release [7]. *unc-31(e928) *mutants are very inactive – almost paralyzed – on food. The suppressor strain KY5029 moves well, in fact it is slightly hyperactive. The strain KY5029 was crossed to Hawaiian males. Heterozygous F1 progeny were singled to plates. 85 Unc-31 animals were singled from among the F2 progeny. Most of these plates of Unc-31 animals segregated active suppressed animals, demonstrating that the suppressors are recessive. Two relatively active animals from each of the 85 plates were singled. From among the 170 plates, 30 plates were found to have the hyperactive phenotype of KY5029. Animals from these 30 plates were combined and used for chromosome mapping. From the chromosome mapping experiment we found that the suppressed *unc-31 *phenotype of KY5029 animals was linked to three genetic regions, on chromosomes I, II, and IV (data not shown). The region on chromosome IV contains *unc-31*, and linkage to IV is expected since Unc-31 animals were selected from among the F2 generation. Thus, the regions on chromosomes I and II must contain mutations suppressing the Unc-31 phenotype.

**Figure 5:**
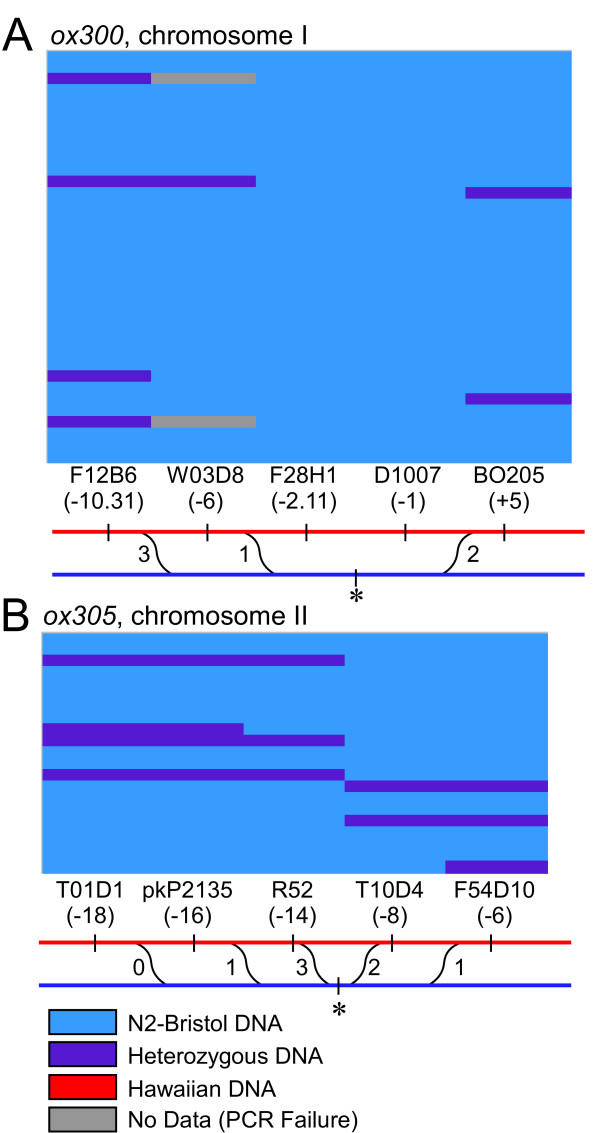
*SNP mapping two synthetic *unc-31 *suppressor mutations*. A, EG5296 *ox300; unc-31(e928) *interval mapping data. B, EG5297 *dpy-5(e61); ox305; unc-31(e928) *interval mapping data. The two suppressor mutations were separated from KY5029 and the suppressing activities were separately mapped to chromosome I and chromosome II, respectively. Each row illustrates the results from a single recombinant animal, and each colored box represents the genotype of a recombinant at the indicated SNP. Bristol is represented by blue, heterozygotes by purple, Hawaiian by red, and PCR failures by gray. F12B6 is wormbase allele snp_F12B6[1], F28H1 is wormbase allele snp_F28H1[1], T10D4 is a polymorphism identified by the St. Louis SNP consortium[5] (see Methods).

We found that the individual suppressor loci are weak *unc-31 *suppressors on their own. From the *unc-31(e928) *plates we singled animals that were less strongly suppressed; specifically, they were slightly sluggish rather than hyperactive. Linkage to the Bristol N2 genotype in these animals was observed on chromosomes I and IV, while linkage to the Hawaiian genotype was observed on chromosome II (data not shown). Thus, chromosome I contains a novel suppressor of *unc-31 *that partially suppresses the *unc-31(e928) *phenotype. Chromosome II contains a mutation that, in combination with the mutation on chromosome I, suppresses the *unc-31(e928) *phenotype to produce hyperactive worms. Further analysis determined that the suppressor on II could also partially suppress *unc-31(e928) *on its own. To simplify future mapping experiments we mapped the independent suppressing activities of the mutations on chromosome I and II in the *unc-31(e928) *background. Both suppressors were then fine mapped using primer sets on I and II independently to the genetic intervals depicted in Figure 5A and 5B. Together, these data suggest that KY5029 contains two mutant loci in addition to *unc-31(e928)*. These complex interactions were deciphered with a minimum of time, effort, and confusion.

## Conclusion

In summary, these methods comprise an accurate and fast technique for mapping that has advantages over both traditional mapping experiments and over other SNP mapping approaches that have been previously described. Compared to traditional mapping, this technique offers standardized, efficient mapping to small intervals for large numbers of mutations, such as might result from a genetic screen. In addition, it allows the mapping of subtle phenotypes (such as behavior) and complex genotypes (such as suppressor or synthetic mutations).

Compared to other SNP mapping methods, the technique described here occupies a comfortable midpoint between the simple but less efficient method described by Wicks *et al*. [1], and the high-throughput but complex techniques published by Swan *et al*. [3] and Zipperlen *et al*. [4]. Although the SNP detection technique described by Wicks is simple and inexpensive, it requires setting up a number of individual PCR reactions and matching the correct PCR product with the correct restriction enzyme. In the methods described here, once the primers (in chromosome mapping) or templates (in interval mapping) are arrayed into a plate, reaction components are accurately dispensed automatically and repeatably. Errors in matching restriction enzymes and buffers to primer sets have been eliminated by using *Dra*I for all reactions. Further, we have found that assaying 8 SNPs on each chromosome means that every mutation is linked to multiple SNPs, giving a high level of redundancy. In fact, because of its cost and accuracy advantages, our technique has been successfully applied in an undergraduate teaching lab setting (M. Peters, personal communication).

The fluorescent polarimetry technique described by Swan *et al*., and the indel detection technique described by Zipperlen *et al*., enable high throughput SNP detection in *C. elegans*. However, these techniques require specialized equipment (a fluorescence polarimeter or capillary sequencer) that are not accessible to every laboratory and that require significant operator knowledge. Compared to those SNP mapping approaches, the technique described here is cheaper and more accessible, since it relies on methods that most labs already use.

In this paper, we present a happy medium between previous approaches to SNP mapping in worms. We build upon the simple, inexpensive, accessible and robust restriction digestion SNP detection technique of Wicks. However our primer sets, equipment and techniques substantially reduce user effort relative to the Wicks method, and so provide the efficiency and low error rate of the Swan or Zipperlen technologies.

## Methods

### Chromosome mapping

Hawaiian CB4856 males were crossed into EG1000 *dpy-5(e61) I; rol-6(e187) II; lon-1(e1820) III*. Fifty Dpy animals and fifty non-Dpy animals from among the self-progeny of EG1000/CB4856 heterozygote hermaphrodites were picked into separate tubes, each containing 20 μL single-worm lysis buffer (50 mM KCl, 10 mM Tris pH 8.3, 2.5 mM MgCl_2_, 0.45% IGEPAL CA-630, 0.45% Tween 20, 0.01% (w/v) gelatin, 60 ug/ml proteinase K). A further 96 Dpy animals were picked to individual plates for use in interval mapping (see below). They were lysed by freezing at -80°C followed by incubation at 65°C 1 hour and proteinase was inactivated by incubation at 95°C 15 minutes. The Dpy lysate DNA templates were then added to a PCR master mix containing 424 μL water, 52 μL 10X PCR buffer (10X: 22.5 mM MgCl_2_, 500 mM Tris-HCl, 140 mM (NH_4_)_2_SO_4_, pH 9.2 at 25°C), 10.4 μL 10 mM dNTPs, and 3.12 μL Taq (5 units/μl). A similar mix was made with the 50 non-Mutant animals. 9.8 μL of the mutant mix or the non-mutant mix was aliquoted into alternate rows of a 96-well PCR plate (Figure 1A). Primer pairs were arrayed into a microtiter plate at 10 μM each primer, so that neighboring rows contain duplicate pairs, and pin-replicated into the master mix. PCR reactions were done using the cycling conditions: 2' 94°C, 35 cycles of (15" 94°C, 45" 60°C, 1' 72°C), 5' 72°C. After amplification, PCR products were digested in the plate with the restriction enzyme *Dra*I in a final volume of 16 μL (10 μL PCR product, 4.15 μL H2O 1.6 μL 10X *Dra*I buffer (New England Biolabs), 0.25 μL *Dra*I (10 units/μL, New England Biolabs)). This was accomplished by adding 6 μL of the enzyme plus enzyme buffer mix to each well using a multi-channel pipette followed by brief centrifugation in a Sorval RT6000D centrifuge with an H1000B rotor. Digestion reactions were incubated at 37°C at least 4 hours. Samples were then loaded onto a 2.5% agarose gel using an 8-channel pipette. The resulting gel displays all 48 SNP markers, from left to right and from chromosome I to X. Each Mutant SNP is next to its non-Mutant control, so that the whole genome can be quickly scanned for linkage.

### Interval mapping

PCR templates were generated by cloning mutant animals from among the self-progeny of EG1000/CB4856 F_1 _hermaphrodites (described above) onto individual seeded plates. After 5 days, self progeny were washed from each plate using water (>100 worms / plate) and placed in a single well of a 96-well plate. Worms were allowed to settle to the bottom of the wells for 15' at 4°C then excess water was pipetted off to leave 45 μl in each well. The plates were frozen and stored at -80°C. The plates were thawed and 15 μl of 4X lysis buffer (200 mM KCl, 40 mM Tris pH 8.3, 10 mM MgCl_2_, 1.8% IGEPAL CA-630, 1.8% Tween 20, 0.04% (w/v) gelatin, 240 ug/ml proteinase K) was then added to each well to give 1X lysis buffer. The plates were covered with sealing tape and briefly vortexed to break up the worm pellet. The worms were lysed by incubation at 65°C 1 hour and 95°C 15 minutes. These PCR templates were stored frozen at -80°C and thawed prior to each use. For each PCR, each well of the 96-well plate received 9.8 μL of a PCR mix containing 8.5 μL water, 1 μL 10X buffer, 0.2 μL 10 mM dNTP, 0.02 μL each primer (100 μM), and 0.06 μL Taq (5 units/μl). Templates were then pin-replicated from the lysis plate. PCR conditions and *Dra*I digests were the same as in chromosome mapping.

### *unc-31(e928) *suppression mapping

We cloned 85 animals with an *unc-31(e928)-*like phenotype from the self progeny of KY5029/CB4586 hermaphrodites. *unc-31(e928) *animals are lethargic and uncoordinated; however, most animals exhibit periodic moments of coordinated movement making it difficult to distinguish between plates with no suppressed progeny and plates with weakly suppressed progeny. Therefore, two animals exhibiting coordinated movement were cloned from each plate. We scored the progeny of these 170 animals and divided them into five classes: uncoordinated, sluggish yet coordinated, coordinated, hyperactive, and mixed. For the sluggish and hyperactive phenotypes, we collected animals for chromosome mapping by combining two animals from each plate. Chromosome mapping was performed as described above, suggesting the presence of two suppressors located on chromosomes I and II (data not shown). To confirm the mapping results, individual recombinants were assayed for SNPs on chromosomes I, II, and IV (data not shown). To simplify further interval mapping experiments these suppressors were crossed away form each other to generate two partially suppressed strains, EG5296 *ox300; unc-31(e928) *and EG5297 *dpy-5(e61); ox305; unc-31(e928)*. For EG5297, we verified the loss of the *ox300 *chromosome by homozygosing a *dpy-5(e61) *marker. Interval mapping of *ox300 *was carried out by cloning 11 *unc-31(e928)*-like self progeny from EG5296/CB4586 hermaphrodites. From these self-progeny 35 sluggish yet coordinated animals were cloned and assayed at SNPs flanking the suppressor (Figure 5A). *ox300 *is located on Chromosome I between W03D8 and B0205. *ox305 *was mapped by cloning 8 *unc-31(e928)*-like self progeny from EG5297/CB4586 hermaphrodites. From these self-progeny 21 partially suppressed animals were cloned and assayed at SNPs flanking the suppressor (Figure 5B). The suppressor in *ox305 *is located on Chromosome II between R52 and T10D4. Four new SNPs that are not part of the chromosome mapping set were used. Wormbase allele snp_F12B6[1] was amplified with primers 5'-caggttggtttttggcaagt-3' 5'-tgattgaacatatccggcaa -3' and detected with *Mfe*I. Wormbase allele snp_F28H1[1] was amplified with primers 5'-gcagtaggcaagagtcaggc-3' and 5'-tattgcacttggctcacagc -3' and was detected with *HpyCH4*V. pkP2135 was amplified with primers 5'-tttgcagatttccgatactgtg -3' and 5'-ttttgtcgtaagacctttggtg -3' and was detected with *Dra*I. A polymorphism on T10D4, referenced at the web page [8] was amplified with primers 5'-gtacgcctcaaaaagtggag -3' and 5'-accacccaacacaatctctg -3' and was detected with *Mse*I.

## Abbreviations used

SNP: Single nucleotide polymorphism.

## Authors' contributions

MWD MH conceived and designed the SNP methods, carried out and supervised the experiments and drafted the manuscript. MWD wrote the scripts that extracted and formatted the SNP data. TH and SO tested SNP primers PH carried out the *unc-31 *mapping experiments. EMJ helped coordinate the experiments and draft the manuscript. All authors read and approved the final manuscript.
